# Disrupted Topological Organization in White Matter Networks in Unilateral Sudden Sensorineural Hearing Loss

**DOI:** 10.3389/fnins.2021.666651

**Published:** 2021-07-12

**Authors:** Yan Zou, Hui Ma, Bo Liu, Dan Li, Dingxi Liu, Xinrong Wang, Siqi Wang, Wenliang Fan, Ping Han

**Affiliations:** ^1^Department of Radiology, Union Hospital, Tongji Medical College, Huazhong University of Science and Technology, Wuhan, China; ^2^Hubei Key Laboratory of Molecular Imaging, Union Hospital, Tongji Medical College, Huazhong University of Science and Technology, Wuhan, China; ^3^Department of Otorhinolaryngology, Union Hospital, Tongji Medical College, Huazhong University of Science and Technology, Wuhan, China; ^4^GE Healthcare, Shanghai, China

**Keywords:** diffusional tensor imaging, deterministic tractography, graph theory, small-worldness, unilateral sudden sensorineural hearing loss

## Abstract

Sudden sensorineural hearing loss (SSNHL) is a sudden-onset hearing impairment that rapidly develops within 72 h and is mostly unilateral. Only a few patients can be identified with a defined cause by routine clinical examinations. Recently, some studies have shown that unilateral SSNHL is associated with alterations in the central nervous system. However, little is known about the topological organization of white matter (WM) networks in unilateral SSNHL patients in the acute phase. In this study, 145 patients with SSNHL and 91 age-, gender-, and education-matched healthy controls were evaluated using diffusion tensor imaging (DTI) and graph theoretical approaches. The topological properties of WM networks, including global and nodal parameters, were investigated. At the global level, SSNHL patients displayed decreased clustering coefficient, local efficiency, global efficiency, normalized clustering coefficient, normalized characteristic path length, and small-worldness and increased characteristic path length (*p* < 0.05) compared with healthy controls. At the nodal level, altered nodal centralities in brain regions involved the auditory network, visual network, attention network, default mode network (DMN), sensorimotor network, and subcortical network (*p* < 0.05, Bonferroni corrected). These findings indicate a shift of the WM network topology in SSNHL patients toward randomization, which is characterized by decreased global network integration and segregation and is reflected by decreased global connectivity and altered nodal centralities. This study could help us understand the potential pathophysiology of unilateral SSNHL.

## Introduction

Sudden sensorineural hearing loss (SSNHL) is a sudden-onset and rapidly developed hearing impairment of at least 30 dB in no less than three contiguous frequencies within 72 h, which is a common emergency in ear-nose-throat clinics, along with tinnitus, vertigo, and so on (Stachler et al., 2012). More than 95% of SSNHL cases are unilateral with no side of preference. The incidence and prevalence of SSNHL is 5–160 cases per 100,000 per year (Schreiber et al., 2010). Due to its ambiguous etiology and pathogenesis, the majority of SSNHL cannot be determined with a defined cause for hearing loss by routine clinical examinations; therefore, these cases are considered as idiopathic SSNHL. At present, the main treatment for SSNHL is steroid therapy (Jung et al., 2016). Unfortunately, the hearing function of patients with SSNHL shows little improvement even after suitable high-quality therapy, which may negatively affect economic indicators for the society and result in the poor long-term quality of their life (Manno et al., 2021).

Previous functional MRI studies have suggested that SSNHL is associated with abnormal functional networks. Xu et al. (2016) indicated abnormal changes in global and nodal properties of functional networks in the acute stage of SSNHL. Using positron emission tomography (PET), Micarelli et al. (2017) revealed hypometabolism in the contralateral auditory cortex in SSNHL patients. Moreover, using electroencephalogram (EEG), Cai et al. (2019) discovered that alterations of central neural networks occurred in the early stage of SSNHL. As brain functional connectivity is influenced and constrained by anatomical connectivity patterns (Honey et al., 2009), anatomical substrates of SSNHL should be investigated to fully understand SSNHL pathophysiology. Furthermore, pathological abnormalities are related to the alterations in brain network topography (Fornito and Bullmore, 2015). In addition, Wang et al. (2014) found that the deficiency of unilateral sensory input changed the activity in sensory regions and reorganized the cognitive control network. Long-term unilateral SSNHL patients have abnormal alterations in the default mode network (DMN) (Zhang et al., 2015), which might influence their cognitive abilities. Whether these alterations occur in the acute phase of SSNHL is unclear. Therefore, it is important to investigate the alterations of anatomical connectivity in SSNHL in the early stage.

As a non-invasive advanced diffusional MRI technique, diffusion tensor imaging (DTI) is widely used to characterize the microstructure of human biological tissues. Additionally, DTI makes it possible to virtually reconstruct white matter (WM) tracts according to the principal directions of water molecule diffusion, which further allows us to model the human brain as a complex network *in vivo* (Bullmore and Sporns, 2009). Then, graph theory can be applied to characterize topological architectures such as small-worldness and hub regions of whole brain WM networks (Rubinov and Sporns, 2010). DTI provides evidence of WM structural alterations in unilateral hearing loss (Wu et al., 2009; Rachakonda et al., 2014). Using region of interest (ROI) methods, altered fractional anisotropy (FA) is involved in the auditory nerve (Vos et al., 2015; Zhang et al., 2020), lateral lemniscus, and inferior colliculus (Lin et al., 2008). Using tract-based spatial statistics analyses, extensive alterations in both auditory and non-auditory tracts and regions were revealed (Shang et al., 2018). However, the nature of these changes in structural connectivity among regions remains unclear, and there is no study to date that has investigated topological changes in the whole brain network of unilateral SSNHL in the acute phase. Analyzing the whole brain network at the integrated level might provide a more comprehensive understanding of brain network abnormalities that might be imperfectly detected by traditional regional or voxel-based analysis (Li et al., 2018). Therefore, it is necessary to evaluate the alterations of global and nodal network parameters of SSNHL to provide a structural basis for the extensive brain reorganization in the WM network caused by early unilateral hearing deprivation.

In this study, graph theoretical analysis methods based on DTI were applied to compare the topological properties of brain WM structural networks at global and nodal levels in unilateral idiopathic SSNHL patients and normal hearing healthy controls (HCs). We hypothesized that disrupted topological organization would be characterized by abnormal global metrics of WM networks in patients with SSNHL in the acute phase. In addition, we hypothesized that SSNHL patient would exhibit abnormal nodal metrics in WM networks, including brain regions beyond the auditory network, such as the DMN and subcortical network.

## Materials and Methods

### Participants

This study was approved by the Ethics Committee of Tongji Medical College of Huazhong University of Science and Technology and was performed in accordance with the Helsinki Declaration. Informed consent was obtained from each subject before study enrollment.

One hundred forty-five patients (76 male, 69 female) with SSNHL were recruited from the ENT Clinic, Wuhan Union Hospital, which is affiliated to Tongji Medical College, Huazhong University of Science and Technology, and 91 age-, gender-, and education-matched HCs (43 male, 48 female) were recruited from the Radiology Department of Wuhan Union Hospital from 2018 to 2019. All the participants were right-handed Han Chinese. Detailed criteria for inclusion in the study were as follows: (1) patients had at least 30 dB sensorineural hearing impairment in no less than three consecutive audiometric frequencies within 72 h. (2) Patients had idiopathic SSNHL (the cause of hearing loss is still unclear after regular clinical and MR examinations). (3) The duration of hearing loss was within 2 weeks. (4) All SSNHL patients had not been treated. (5) HCs had normal hearing (not more than 25 dB). Detailed criteria for exclusion were as follows: (1) all subjects had neurological diseases (such as acoustic neuroma, brain tumors, and trauma), psychiatric diseases (such as depression and insomnia), and other otolaryngological diseases (such as otitis media, Meniere disease, and pulsatile tinnitus); and (2) all subjects had claustrophobia and cardiac pacemakers.

### Pure Tone Audiometry

Both SSNHL patients and HC subjects underwent pure tone audiometry (PTA) tests. Air conduction thresholds were measured at 125, 250, 500, 1,000, 2,000, 4,000, and 8,000 Hz for both ears, and bone conduction thresholds were measured between 250 and 4,000 Hz in a soundproof room. Moreover, the average hearing threshold at the frequencies of 0.5, 1, 2, and 4 kHz was defined as the mean hearing threshold (Micarelli et al., 2017).

### Tinnitus Assessment

Due to more than 80% of SSNHL patients complaining of tinnitus, the Tinnitus Handicap Inventory (THI) was applied to evaluate tinnitus severity, according to an increasing handicap scale ranging from 0 to 100 (Newman et al., 1996).

### Image Acquisition

All SSNHL and HC subjects underwent the same imaging protocols performed on a 3.0-T scanner (Siemens Trio Tim, Erlangen, Germany) that was equipped with a 12-channel head coil. Participants were asked to lay still in a supine position. Foam pads were used to fix their heads to reduce head motion during scanning. Three-dimensional (3D) high-resolution T1-weighted images were acquired with the following scanning parameters: repetition time (TR), 2,250 ms; echo time (TE), 2.26 ms; inversion time (TI), 900 ms; flip angle, 9°; voxel size, 1.0 × 1.0 × 1.0 mm^3^; field of view (FOV), 256 mm × 256 mm; matrix dimension, 256 × 256; slice thickness, 1 mm; and 176 sagittal slices covering the whole brain. DTI was obtained using a single shot spin-echo-planar sequence with *b* values of 0 and 1,000 s/mm^2^; 30 non-linear diffusion-weighting gradient directions with the following scanning parameters were used: TR, 6,000 ms; TE, 93 ms; flip angle, 90°; FOV, 200 mm × 200 mm; matrix dimension, 128 × 128; slice thickness, 2 mm; and 44 slices covering the whole brain. Individuals with abnormal MR signals (such as acoustic neuroma and brain tumors) were excluded.

### Image Preprocessing

Diffusion tensor imaging preprocessing and WM network construction were conducted using PANDA software (^1^ a pipeline tool for diffusion MRI analysis; Cui et al., 2013). Preprocessing steps included the following: (1) eddy current and motion artifact correction of the DTI data were performed with FSL^2^. (2) Skull removal was done using brain extraction tool. (3) The diffusion tensor models were built by the linear least squares fitting method at each voxel using diffusion toolkit^3^. (4) For each subject, whole brain deterministic tractography was performed in native diffusion space by fiber assignment by continuous tracking (FACT) algorithm with diffusion toolkit. All of the tracts in the dataset were calculated by seeding each voxel with FA greater than 0.2. Tractography was terminated if the angle was more than 45° or if a voxel with an FA < 0.2 was observed (Hu et al., 2020).

### Network Construction

**FIGURE 1:**
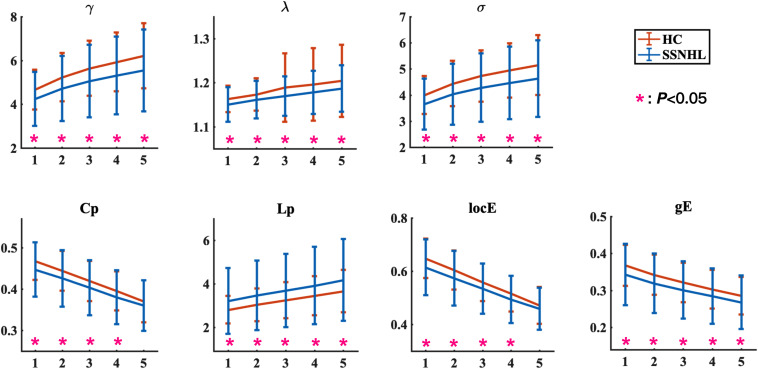
Global network parameters in HCs and patients with SSNHL over the selected FN thresholds. FN = *n* (1, 2, 3, 4, 5) indicates that at least streamlines must be present for a link to be drawn. The black stars (*) indicate a significant between-group difference (*p* < 0.05). Bars and error bars indicate mean values and standard deviation, respectively. Both the HC group and the SSNHL group showed γ greater than 1, λ approximately equal to 1, and σ greater than 1, which indicates that the WM network of both groups exhibited a small-world topology. γ, normalized clustering coefficient; λ, normalized characteristic path length; σ, small-worldness; Cp, clustering coefficient; Lp, characteristic path length; locE, local efficiency; gE, global efficiency; HC, healthy control; SSNHL, sudden sensorineural hearing loss.

The WM networks were constructed at the macroscale level with nodes representing brain regions and edges representing structural connectivity between brain regions (Kang et al., 2017). To define nodes of the WM network, the automated anatomical labeling (AAL) atlas was applied to divide the whole brain into 90 regions (Supplementary Table 1; Tzourio-Mazoyer et al., 2002). For each subject, the T1-weighted image was coregistered to the b0 image in native diffusion space using a linear transformation, and then non-linearly transformed to the T1 template in Montreal Neurological Institute (MNI) space. Then, the derived inverse transformation parameters were inverted and used to warp the AAL atlas from MNI space to the native diffusion space in which the discrete labeling values were preserved with a nearest neighbor interpolation method. Then, the individual b0 image and the transformed AAL atlas were observed in the individual space to ensure that there were no obvious mismatching errors. To define the edges of the WM network, structural connectivity was considered to exist if at least three fiber bundles were located between two brain regions (Liu et al., 2017). Moreover, fiber numbers (FNs) ranging from 1 to 5 were set as thresholds to evaluate the effects of different thresholds on the WM network topological properties. We found that different thresholds did not significantly affect our results (Figure 1). Therefore, the results were mainly based on a value of FN = 3 unless otherwise specified. As a result, for each participant, we obtained an unweighted binary network that was represented by a symmetrical anatomical 90 × 90 matrix.

### Network Analysis

Graph theoretical analysis was applied to each participant’s WM network *via* GRETNA software^4^ (Wang et al., 2015). For global topological parameters, we calculated clustering coefficient (Cp), characteristic path length (Lp), local efficiency (locE), global efficiency (gE), normalized clustering coefficient (γ), normalized characteristic path length (λ), and small-worldness (σ).

For nodal parameters, we computed three nodal centrality metrics: nodal degree centrality (Di) (Rubinov and Sporns, 2010), nodal efficiency (Ei) (Achard and Bullmore, 2007), and nodal betweenness centrality (Bi). Additionally, we calculated WM network hubs, which are highly connected nodes and are regarded to have a vital role in a network (Fischer et al., 2019). Specifically, hubs should show high Di, high Bi, low Lp, and low Cp. Therefore, we identified the hubs of WM networks according to the above definition. Each node of 90 anatomically defined brain regions was ranked with a score of one each time any of the following conditions were met (van den Heuvel et al., 2010): (1) the node was in the top 20% of nodes with the highest Di; (2) it was in the top 20% of nodes with the highest Bi; (3) it was in the bottom 20% of nodes with the lowest Cp; or (4) it was in the bottom 20% of nodes with the lowest Lp. The result was a node-specific hubness score that ranged from zero to four. Using this method, we defined the brain regions that had a score of at least of two as the putative hub regions of the anatomical connectome.

### Statistical Analysis

The group differences in age, education level, and PTA were analyzed using two-tailed independent-sample *t-*test. The group difference in gender was analyzed using χ^2^ test. Pairwise comparisons were performed using a general linear model to determine the between-group differences in global network parameters and regional nodal centrality with regressing age, gender, and education level. In addition, Bonferroni correction was performed to control for the error of multiple comparisons in 90 nodes in nodal network properties. *p*-value ≤ 0.05 was considered statistically significant.

Pearson’s correlation analyses were assessed to indicate the relationship between network parameters with significant group differences and clinical variables with age, gender, and education level as unconcerned confounding factors.

## Results

### Demographic and Clinical Data

Demographic and clinical data for all the participants are shown in Table 1. No significant differences in age, gender, or education level (all *p* > 0.05) were found between SSNHL patients and HCs. PTA between the two groups was significantly different (*p* < 0.001).

**TABLE 1:** Demographic and clinical data.

Demographic	HC	SSNHL	*p-*value
Number (*n*)	91	145	N/A
Gender (male/female)	43/48	76/69	0.504^*a*^
Age (years)	38.48 ± 12.66	38.51 ± 11.65	0.987^*b*^
Handedness (left/right)	0/91	0/145	N/A
Education (years)	13.33 ± 3.79	12.73 ± 3.24	0.224^*b*^
Duration (days)	N/A	8.44 ± 4.17	N/A
Effected ear (left/right)	N/A	75/70	N/A
PTA (dBHL)	12.08 ± 4.04	78.17 ± 28.56	<0.0001^*b*^
THI score (0–100)	N/A	45.57 ± 24.88	N/A

*Values are represented as the mean ± SD. PTA of HC was the average PTA value of the bilateral ears. ^*a*^*p*-value was obtained using Pearson χ^2^ test (two-tailed). ^*b*^*p*-value was obtained using the independent-sample *t-*test (two-tailed). HC, healthy control; SSNHL, sudden sensorineural hearing loss; PTA, pure tone average; THI, tinnitus handicap inventory.*

### Global Parameters

The overall means of group global topological organization (λ, γ, σ, Cp, locE, gE, and Lp) are shown as a function of the threshold from *n* = 1∼5, where *n* represents the minimum number of WM fibers needed to construct a connection between a pair of nodes.

**FIGURE 2:**
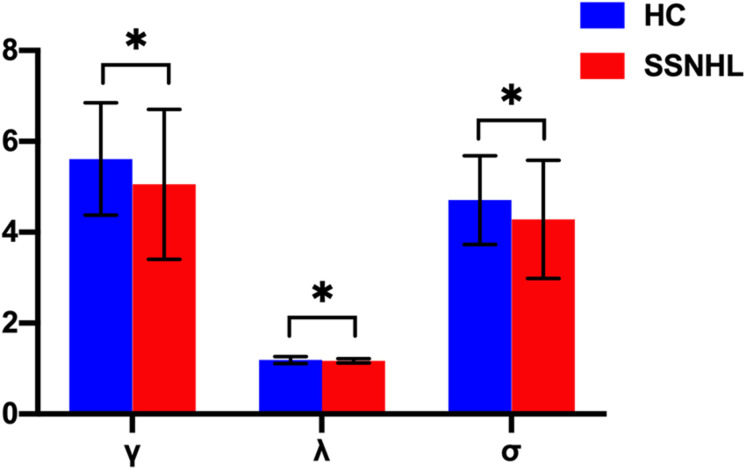
Between-group differences in small-world properties between SSNHL and HC groups. The black stars (^∗^) indicate a significant between-group difference (*p* < 0.05). Error bars represent standard deviations. γ, normalized clustering coefficient; λ, normalized characteristic path length; σ, small-worldness; HC, healthy control; SSNHL, sudden sensorineural hearing loss.

**FIGURE 3:**
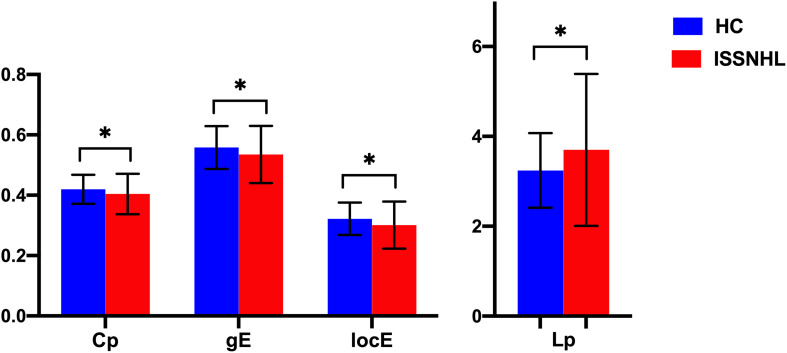
Between-group differences in global parameters between the SSNHL and HC groups. The black stars (^∗^) indicate a significant between-group difference (*p* < 0.05). Error bars represent standard deviations. Cp, clustering coefficient; gE, global efficiency; locE, local efficiency; Lp, characteristic path length; HC, healthy control; SSNHL, sudden sensorineural hearing loss.

Both the SSNHL and HC groups showed a small-world organization of the WM connectivity networks, as expressed by γ≫ 1, λ≈ 1, σ > 1 (Table 2 and Figure 1). Compared with the HC group, SSNHL patients showed significantly decreased γ, λ, σ, Cp, locE, and gE and significantly increased Lp (Figures 2, 3; *p* < 0.05).

**TABLE 2:** Between-group differences in global parameters between the SSNHL and HC groups.

Global parameters	HC (*n* = 91)	SSNHL (*n* = 145)	*T*-value	*p*-value
γ	5.613 ± 1.239	5.054 ± 1.650	2.815	0.005
λ	1.189 ± 0.077	1.170 ± 0.045	2.449	0.015
σ	4.707 ± 0.974	4.283 ± 1.301	2.717	0.007
Cp	0.420 ± 0.048	0.404 ± 0.067	1.984	0.048
Lp	3.242 ± 0.830	3.695 ± 1.692	−2.279	0.018
locE	0.558 ± 0.071	0.535 ± 0.095	2.068	0.040
gE	0.322 ± 0.054	0.301 ± 0.078	2.201	0.029

*Comparisons were performed using a general linear model to determine the between-group differences in global network parameters with age, gender, and education level as covariates. γ, normalized clustering coefficient; λ, normalized characteristic path length; σ, small-worldness; Cp, clustering coefficient; Lp, characteristic path length; locE, local efficiency; gE, global efficiency; HC, healthy control; SSNHL, sudden sensorineural hearing loss.*

### Regional Parameters

**FIGURE 4:**
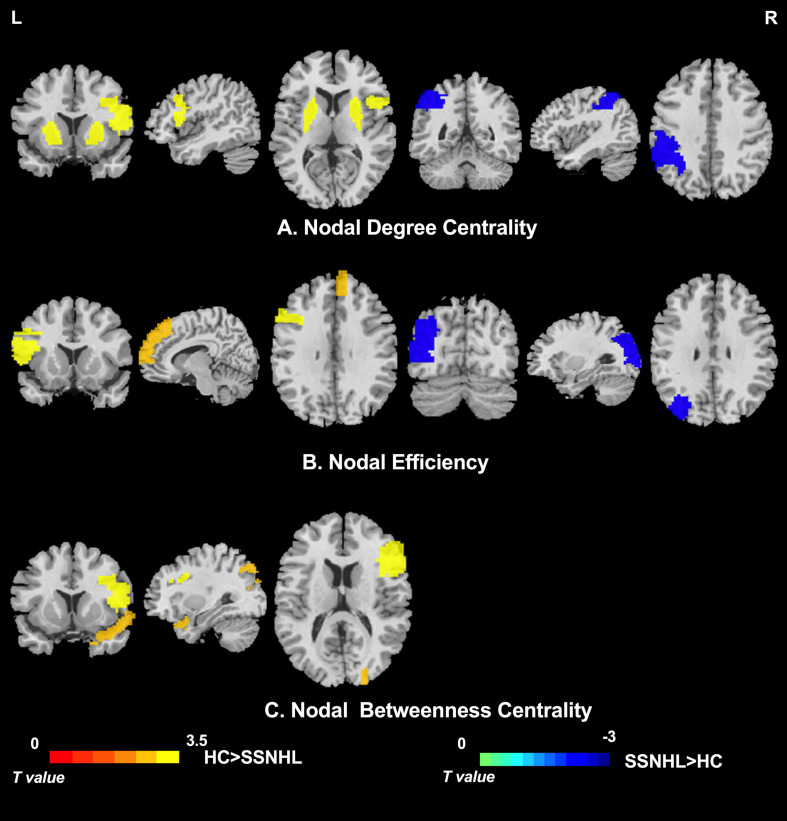
Between-group differences in nodal centrality of brain regions between the SSNHL and HC groups (*p* < 0.05, Bonferroni corrected). **(A)** Between-group differences in nodal degree centrality; **(B)** between-group differences in nodal efficiency; and **(C)** between-group differences in nodal betweenness centrality. The color bar indicates the value of *T*. See Table 3 for more details. HC, healthy control; SSNHL, sudden sensorineural hearing loss; L, left; R, right.

As displayed in Table 3, significant differences in nodal centralities (Di, Ei, and Bi) in many brain regions were noted between the HC and SSNHL groups (*p* < 0.05, Bonferroni corrected). Compared with HCs, SSNHL patients exhibited reduced Di in the right inferior frontal gyrus, opercular part (IFGoperc.R), left putamen (PUT.L), and right putamen (PUT.R); increased Di in the left inferior parietal (IPL.L) (Figure 4A); reduced Ei in the left inferior frontal gyrus (IFGoperc.L) and right medial superior frontal gyrus (SFGmed.R); increased Ei in the left middle occipital gyrus (MOG.L) (Figure 4B); and reduced Bi in the IFGoperc.R, right inferior frontal gyrus, triangular part (IFGtriang), and right temporal pole: superior temporal gyrus (TPOsup.R) (Figure 4C). These altered brain regions were predominantly located in the auditory network (AUN), visual network (VN), DMN, attention network, sensorimotor network (SMN), and subcortical network.

**TABLE 3:** Between-group differences in nodal parameters between the SSNHL and HC groups.

Brain region	Di	Ei	Bi
**HC > SSNHL**			
IFGoperc.L	0.0481	0.0015**	0.9950
IFGoperc.R	0.0011**	0.0065	0.0007**
IFGtriang.R	0.0444	0.1512	0.0016**
SFGmed.R	0.7851	0.0043**	0.1928
SOG.R	0.2350	0.3598	0.0038**
PUT.L	0.0017**	0.1106	0.0059
PUT.R	0.0022**	0.0116	0.0121
TPOsup.R	0.0128	0.1695	0.0047**
**SSNHL > HC**			
MOG.L	0.2794	0.0036**	0.0378
IPL.L	0.0039**	0.1650	0.0915

The black stars (**) indicate a significant between-group difference (*p* < 0.05, Bonferroni corrected). All regional abbreviations can be found in Supplementary Table 1. HC, healthy control; SSNHL, sudden sensorineural hearing loss; Di, nodal degree centrality; Ei, nodal efficiency; Bi, nodal betweenness centrality.

**FIGURE 5:**
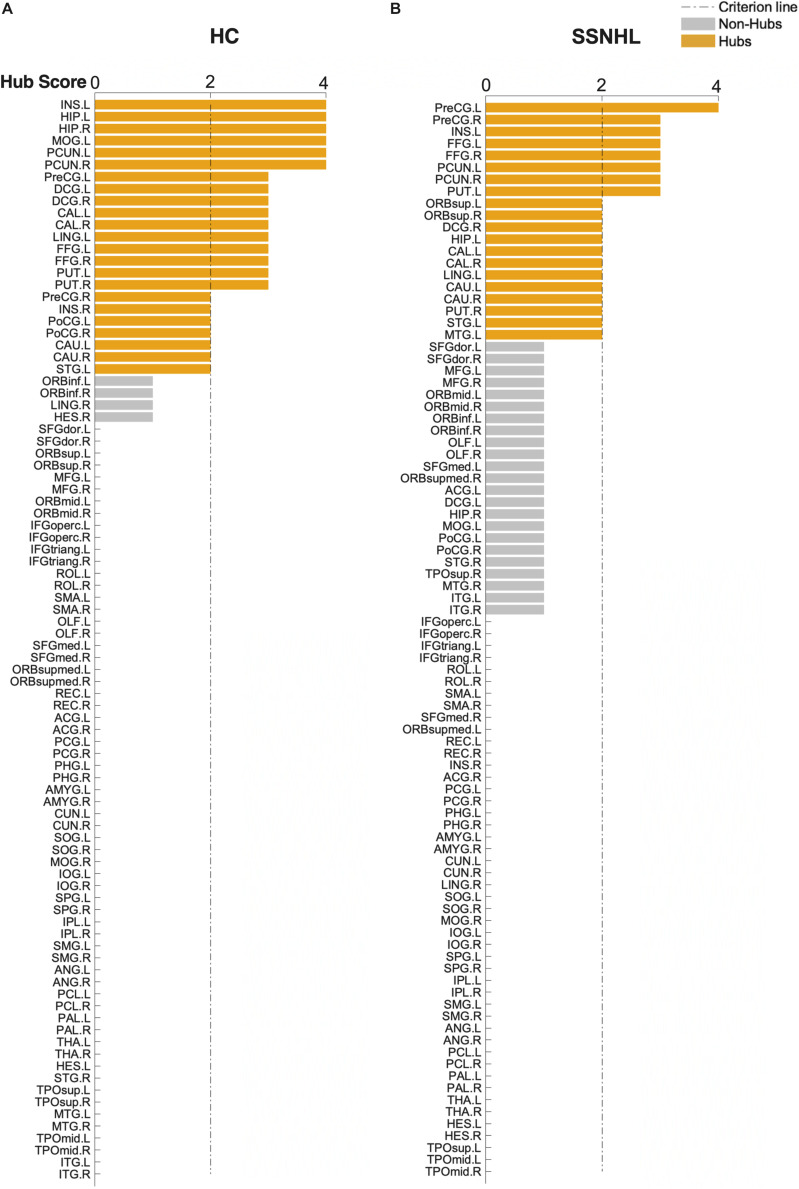
Hub score for the HC and SSNHL groups. **(A)** Hub regions for the HC group; **(B)** hub regions for the SSNHL group. According to the criteria that were mentioned previously, 90 brain regions were scored and sorted. A score ≥ 2 indicated hub regions (yellow) and a score < 2 indicated non-hub regions (gray). Definitions of all regional abbreviations can be found in Supplementary Table 1. HC, healthy control; SSNHL, sudden sensorineural hearing loss.

**FIGURE 6:**
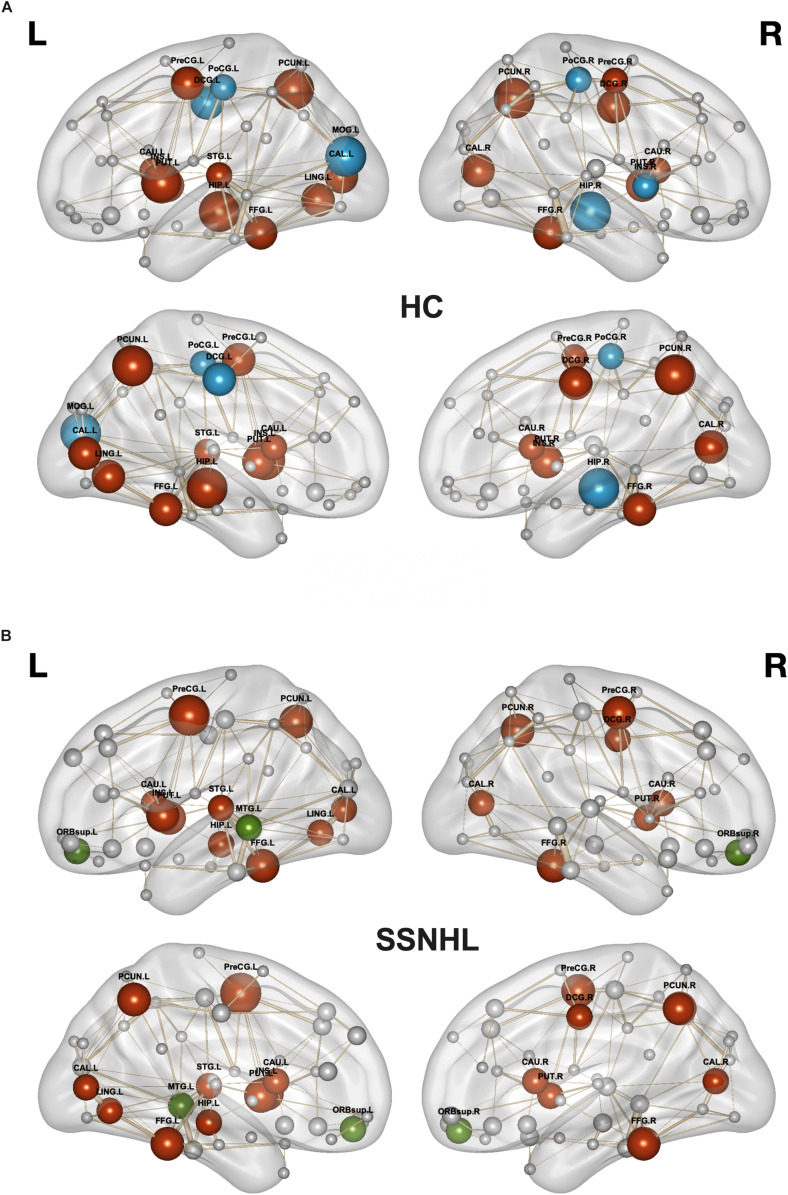
Hub regions for HCs and SSNHL patients. **(A)** Hub regions for the HC group; **(B)** hub regions for the SSNHL group. Blue nodes represent hubs specific to HCs; green nodes represent hubs specific to SSNHL patients; red nodes represent shared hubs in the two groups; gray nodes represent non-hubs. The sizes of the nodes represent the hub scores. Definitions of all regional abbreviations can be found in Supplementary Table 1. HC, healthy control; SSNHL, sudden sensorineural hearing loss.

As displayed in Table 4 and Figure 5, brain hub regions of WM networks for the two groups were identified according to the criterion (score ≥ 2) that was mentioned previously. Twenty-three hub regions were found in HCs, and 20 hub regions were found in SSNHL patients. Among these hubs, most hubs were shared in the both groups; hubs specific to HC were located in the AUN, VN and subcortical network; hubs specific to SSNHL were belong to the DMN (Figure 6).

**TABLE 4:** Brain hub regions of WM networks in the HC and SSNHL groups.

Group	Brain hub regions	Classification
Shared hubs in the two groups	PreCG.L	Primary
	PreCG.R	Primary
	INS.L	Paralimbic
	DCG.R	Paralimbic
	HIP.L	Limbic
	CAL.L	Primary
	CAL.R	Primary
	LING.L	Association
	FFG.L	Association
	FFG.R	Association
	PCUN.L	Association
	PCUN.R	Association
	CAU.L	Subcortical
	CAU.R	Subcortical
	PUT.L	Subcortical
	PUT.R	Subcortical
	STG.L	Association
Hubs specific to HC	INS.R	Paralimbic
	DCG.L	Paralimbic
	HIP.R	Limbic
	MOG.L	Association
	PoCG.L	Primary
	PoCG.R	Primary
Hubs specific to SSNHL	ORBsup.L	Paralimbic
	ORBsup.R	Paralimbic
	MTG.L	Association

*All regional abbreviations can be found in Supplementary Table 1. HC, healthy control; SSNHL, sudden sensorineural hearing loss.*

### Relationships Between Topological Properties and Clinical Variables

Within the SSNHL groups, correlations between the altered global or nodal parameters and the clinical variables (such as the duration of hearing loss, PTA, and THI scores) were performed. We found a slightly negative correlation between the λ and the duration of hearing loss (*r* = −0.182, *p* = 0.028) as well as a slightly positive correlation between λ and THI scores (*r* = 0.181, *p* = 0.029) in SSNHL patients (Supplementary Figure 1). In addition, the correlations between the altered nodal properties of the WM network and clinical data are listed in Supplementary Table 2.

## Discussion

To the best of our knowledge, few studies have aimed to explore the whole brain topological pattern of the anatomical connectome of SSNHL in the acute phase by using deterministic tractography and graph analysis. In this study, we recruited 145 unilateral idiopathic SSNHL patients in the acute phase without therapy. The topological architectures of the WM network in SSNHL patients were investigated. Compared with HCs, SSNHL patients showed disrupted topological convergent and divergent patterns of the WM network, which was indicated by reduced γ, λ, σ, Cp, locE, and gE and increased Lp. Additionally, compared with HCs, SSNHL patients had altered nodal centralities in the AUN, VN, DMN, attention network, SMN, and subcortical network.

The human brain is considered to be a complex, small-world network (Sporns and Honey, 2006) that exhibits a balance between network integration (λ, Lp, and gE) and network segregation (γ, Cp, and locE) of information processing. Despite having a small-world topology similar to HCs, the WM network of SSNHL patients shifts toward randomization with lower γ and σ. This randomization of global properties has been previously reported in some diseases such as posttraumatic stress disorder (Suo et al., 2019), schizophrenia (Xia et al., 2019), and so on. Randomization may represent greater resilience to targeted attacks on network hubs.

The global network parameters in WM networks were altered in SSNHL patients, manifesting as reduced Cp, locE, and gE and increased Lp. Lower Cp and locE represented decreased segregation of WM networks in the SSNHL group, which might result from the decreased nodal centralities of several brain regions. Increased locE was found in patients with long-term single-side hearing loss, indicating enhancement of fiber bundles used for transferring information, which may be a type of compensation for decreased auditory input (Li et al., 2019). Their results differ from ours, which may be due to the different durations of hearing loss. Compensation occurred when the durations were longer. However, in our study, the global network properties of SSNHL patients were mainly characterized by destruction for the time period within 2 weeks. gE reflects the ability to transmit information among remote brain regions and is mainly related to long-range connections (Bai et al., 2012). Thus, lower gE implied less efficient overall local segregation in SSNHL patients, which eventually resulted in impaired information processing (Nigro et al., 2016). In addition, Lp reflects the effectiveness of interregional information transmission. Thus, a higher Lp may be attributed to the degeneration of fiber bundles used for information transmission. Given that gE is inversely proportional to Lp (Rubinov and Sporns, 2010), decreased gE and increased Lp represented disrupted global integration of anatomical connectomes as a result of degeneration of fiber bundles for long-range information dissemination and disconnections between distant brain regions in SSNHL patients. In summary, we infer that global hypoconnectivity in the WM network might be one of the underlying mechanisms for abnormal brain development in SSNHL patients.

Compared with HCs, the altered nodal centralities and hub regions in the acute phase of SSNHL patients involved in AUN, which includes the primary auditory cortex and secondary auditory cortex, are responsible for reception and processing of auditory information. Notably, partial auditory deprivation could lead to deficient myelination of WM fibers in the auditory pathway and alterations of morphology and function in the auditory cortex as well in patients with hearing loss (Heggdal et al., 2016; Qi et al., 2019). We found deceased nodal centralities and altered hubs in the superior temporal gyrus (STG), which might be related to the decline of gray matter volume in STG in unilateral SSNHL patients (Fan et al., 2015).

Schmithorst et al. (2014) found altered activation in VN in children with unilateral hearing loss by using audiovisual task-fMRI. Alterations of regional homogeneity in VN such as bilateral calcarine cortices were observed (Wang et al., 2014). In this study, nodal centrality increased in VN in SSNHL patients compared with HCs. Combined with these above studies, we postulate that a stronger reliance on visual information is necessary due to the decline in sound stimulation in the early stage of unilateral SSNHL patients.

Interaural time differences and intensity differences between bilateral ears are necessary for speech recognition in noise and sound localization in subjects with normal hearing (Harris et al., 2009). For effective auditory communication, a listener must selectively pay attention to the relevant sound signals from a coherent auditory stream in a noisy environment to easily perform the same tasks that normal listeners perform (Lee et al., 2008). However, listeners with single-side hearing loss have great difficulty in filtering out the irrelevant acoustic information. As a result, auditory processing is impaired, and attention is diminished without sufficient information in an auditory stream which is filled in by cognition and memory (Xu et al., 2019).

Changes in DMN were found in both long-term unilateral SSNHL (Heggdal et al., 2016) and acute unilateral SSNHL (Xu et al., 2016). Under normal circumstances, the brain regions of DMN exhibit a higher level of neuronal activity at rest, but are negatively activated in cognitive-related tasks (van den Heuvel and Pol, 2010). The DMN allows the brain to carry out various cognitive functions and is regarded as an indicator of neuropathological mechanisms (Greicius, 2008; Qin et al., 2020). Our study observed that nodal centrality decreased in DMN. Zhang et al. (2015) found that long-term unilateral SSNHL patients contributed to alterations in the DMN, and these alterations might have a negative influence on language learning ability and cognitive abilities in these patients (Schmithorst et al., 2014). In addition, with the prolongation of the duration of hearing loss, the range and degree of the brain nerve damage will continue to progress (Zhang et al., 2020). These results suggested that early treatment is important to prevent acute SSNHL from developing into long-term hearing loss.

In this study, we observed that the nodal attributes were altered in the precentral gyrus (PreCG), which belongs to the SMN. Prior studies of using [^18^F] fluorodeoxyglucose (FDG)-PET demonstrated increased FDG uptake in the PreCG of SSNHL patients in the acute phase. Glucose consumption in the PreCG was found to be positively correlated with speech discrimination scores (Micarelli et al., 2017). Using a rhyme judgment task-fMRI, adults with congenital deafness and dyslexia showed greater activation in the PreCG compared with non-dyslexic hearing individuals (MacSweeney et al., 2009). Connections with PreCG were also found to be decreased in patients with unilateral SSNHL whose illness duration was approximately 3 years (Zhang et al., 2018). Taken together, these results indicate that SMN might be involved in word recognition and phonological processing.

The subcortical structure includes the hippocampus, olfactory cortex, and amygdala; these structures belong to the limbic network and are components of the Papez loop. Furthermore, the limbic network is connected with the thalamus and cerebral cortex and is involved in advanced brain functions such as motion, emotion, behavior, learning, and memory (Morgane et al., 2005). In this study, the THI score was slightly correlated with the Bi of the subcortical network in SSNHL patients, which suggested that the decline in unilateral hearing perception may result in the alteration of nodal centrality noted in the subcortical network in SSNHL patients. In addition, several studies have reported that the limbic network is responsible for the signal processing of tinnitus based on the “noise cancelation” mechanism. When the limbic network is compromised, tinnitus can be perceived by patients (Chen et al., 2017). Most SSNHL patients have tinnitus, which might be one of the mechanisms that cause abnormalities in the subcortical network in SSNHL.

This study had several limitations. First, this study was cross-sectional. The manner in which the WM network architecture associated with SSNHL evolves dynamically and can be used to predict the prognosis after therapy remains to be clarified in longitudinal studies. We plan to predict hearing outcomes in SSNHL patients *via* machine learning models and WM network parameters by evaluating PTA of SSNHL after 3 months of follow-up. Second, as DTI is associated with restrictions in the resolution of crossing fibers, diffusion spectrum imaging, which can overcome these difficulties, will be applied to explore the WM network of SSNHL in our future study. Third, although the AAL atlas is still widely applied to investigate human brain organizations, regions with a variety of sizes in AAL may have an influence on nodal centrality (Zhong et al., 2015). In further studies, we will apply more templates to determine which brain parcellation strategy is most appropriate for characterizing WM network topology in unilateral SSNHL.

## Conclusion

Our research revealed disrupted topological organization of the WM network at both global and regional levels in idiopathic unilateral SSNHL patients in the acute period with graph theory analysis. The structural connectome of unilateral SSNHL patients is characterized by a shift toward randomization. We also found altered nodal centralities involved in the AUN and non-auditory networks. These findings could advance our understanding of the potential pathophysiology of unilateral SSNHL. It is our hope that this study could provide additional information about the alteration of whole brain WM networks in unilateral SSNHL patients.

## Data Availability Statement

The raw data supporting the conclusions of this article will be made available by the authors, without undue reservation.

## Ethics Statement

The studies involving human participants were reviewed and approved by Ethics Committee of Tongji Medical College of Huazhong University of Science and Technology. The patients/participants provided their written informed consent to participate in this study.

## Author Contributions

YZ, HM, WF, and PH designed the study, supervised the experiments, and wrote the manuscript. BL, DL, DLiu, XW, and SW performed the experimental work and its analysis and refined the manuscript. All authors contributed to the article and approved the submitted version.

## Conflict of Interest

XW was employed by company GE Healthcare, Shanghai. The remaining authors declare that the research was conducted in the absence of any commercial or financial relationships that could be construed as a potential conflict of interest.

## Footnotes

1 http://www.nitrc.org/projects/panda/

2 http://www.fmrib.ox.ac.uk/fsl

3 TrackVis.org

4 http://www.nitrc.org/projects/gretna/
