# Supplementary material for: Disrupted Topological Organization in White Matter Networks in Unilateral Sudden Sensorineural Hearing Loss
Source: Front Neurosci. 2021 Jul 12;15:666651. doi: 10.3389/fnins.2021.666651 (PMC8312563; doi:10.3389/fnins.2021.666651)
Supplement: Supplementary file 1 [file Data_Sheet_1.docx]

**Supplementary data**

**Table 1** **|** Cortical and subcortical regions of interest defined in the study

| **Index** | **Regions** | **Abbreviation** | **Index** | **Regions** | **Abbreviation** |
| --- | --- | --- | --- | --- | --- |
| (1,2) | Precental gyrus | PreCG | (47,48) | Lingual gyrus | LING |
| (3,4) | Superior frontal gyrus, dorsolateral | SFGdor | (49,50) | Superior occipital gyrus | SOG |
| (5,6) | Superior frontal gyrus, dorsolateral | ORBsup | (51,52) | Middle occipital gyrus | MOG |
| (7,8) | Middle frontal gyrus | MFG | (53,54) | Inferior occipital gyrus | IOG |
| (9,10) | Middle frontal gyrus, orbital part | ORBmid | (55,56) | Fusiform gyrus | FFG |
| (11,12) | Inferior frontal gyrus, opercular part | IFGoperc | (57,58) | Postcentral gyrus | PoCG |
| (13,14) | Inferior frontal gyrus, triangular part | IFGtriang | (59,60) | Superior parietal gyrus | SPG |
| (15,16) | Inferior frontal gyrus, orbital part | ORBinf | (61,62) | Inferior parietal, but supramarginal and angular gyri | IPL |
| (17,18) | Rolandic operculum | ROL | (63,64) | Supramarginal gyrus | SMG |
| (19,20) | Supplementary motor area | SMA | (65,66) | Angular gyrus | ANG |
| (21,22) | Olfactory cortex | OLF | (67,68) | Precuneus | PCUN |
| (23,24) | Superior frontal gyrus, medial | SFGmed | (69,70) | Paracentral lobule | PCL |
| (25,26) | Superior frontal gyrus, medial orbital | ORBsupmed | (71,72) | Caudate nucleus | CAU |
| (27,28) | Gyrus rectus | REC | (73,74) | Lenticular nucleus, putamen | PUT |
| (29,30) | Insula | INS | (75,76) | Lenticular nucleus, pallidum | PAL |
| (31,32) | Anterior cingulate and paracingulate gyri | ACG | (77,78) | Thalamus | THA |
| (33,34) | Median cingulate and paracingulate gyri | DCG | (79,80) | Heschl gyrus | HES |
| (35,36) | Posterior cingulate gyrus | PCG | (81,82) | Superior temporal gyrus | STG |
| (37,38) | Hippocampus | HIP | (83,84) | Temporal pole: superior temporal gyrus | TPOsup |
| (39,40) | Parahippocampal gyrus | PHG | (85,86) | Middle temporal gyrus | MTG |
| (41,42) | Amygdala | AMYG | (87,88) | Temporal pole: middle temporal gyrus | TPOmid |
| (43,44) | Calcarine fissure and surrounding cortex | CAL | (89,90) | Inferior temporal gyrus | ITG |
| (45,46) | Cuneus | CUN |  |  |  |

**Table 2** **|** Relationships between altered WM network parameters and clinical data

| **Correlation** | **r-value** | ***p*-value** |
| --- | --- | --- |
| duration-Lambda | -0.1879 | 0.0237 |
| PTA-ORBmid.R-Di | 0.1644 | 0.0481 |
| PTA-ORBsupmed.R-Di | 0.1764 | 0.0338 |
| PTA-IPL.L-Di | -0.2119 | 0.0105 |
| PTA-CAU.R-Di | 0.1910 | 0.0214 |
| PTA-PUT.L-Di | 0.1747 | 0.0355 |
| PTA-PUT.R-Di | 0.2197 | 0.0079 |
| PTA-PAL.L-Di | 0.2317 | 0.0050 |
| PTA-PUT.R-Bi | 0.2380 | 0.0039 |
| PTA-ORBinf.L-Ei | 0.1789 | 0.0313 |
| PTA-SFGmed.L-Bi | 0.1824 | 0.0281 |
| PTA-PCUN.R-Ei | 0.2000 | 0.0159 |
| THI-Lambda | 0.1786 | 0.0316 |
| THI-CAU.R-Bi | 0.1892 | 0.0227 |
| THI-PUT.L-Bi | 0.2100 | 0.0112 |
| THI-SPG.L-Ei | -0.1877 | 0.0238 |


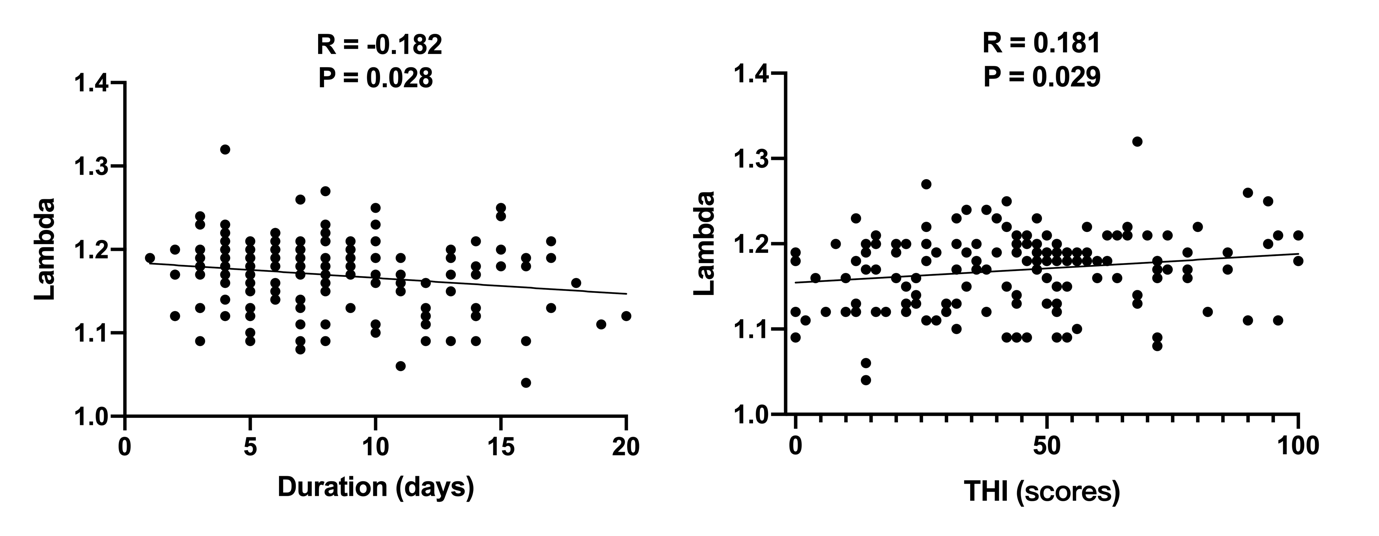
**FIGURE 1** **|** Relationships between altered global WM network parameters and clinical data.
